# Post-Stroke Depression Modulation and *in Vivo* Antioxidant Activity of Gallic Acid and Its Synthetic Derivatives in a Murine Model System

**DOI:** 10.3390/nu8050248

**Published:** 2016-04-28

**Authors:** Seyed Fazel Nabavi, Solomon Habtemariam, Arianna Di Lorenzo, Antoni Sureda, Sedigheh Khanjani, Seyed Mohammad Nabavi, Maria Daglia

**Affiliations:** 1 Applied Biotechnology Research Center, Baqiyatallah University of Medical Sciences, P.O. Box 19395-5487, Tehran 19395-5487, Iran; Nabavisf@gmail.com (S.F.N.); Nabavi208@gmail.com (S.M.N.); 2 Pharmacognosy Research Laboratories, Medway School of Science, University of Greenwich, Chatham-Maritime, Kent ME4 4TB, UK; s.habtemariam@herbalanalysis.co.uk; 3 Department of Drug Sciences, Medicinal Chemistry and Pharmaceutical Technology Section, Pavia University, Viale Taramelli 12, Pavia 27100, Italy; arianna.dilorenzo01@universitadipavia.it; 4 Grup de Nutrició Comunitària i Estrès Oxidatiu (IUNICS) and CIBEROBN (Physiopathology of Obesity and Nutrition), Universitat de les Illes Balears, Palma de Mallorca E-07122, Spain; tosugo@hotmail.com; 5 Department of Physiology, Faculty of Biological Sciences, Shahid Behshti University, P.O. Box 19615-1178, Tehran 19615-1178, Iran; s.khanjani66@yahoo.com

**Keywords:** depression, gallic acid, ischemia, stroke

## Abstract

Gallic acid (3,4,5-trihydroxybenzoic acid, GA) is a plant secondary metabolite, which shows antioxidant activity and is commonly found in many plant-based foods and beverages. Recent evidence suggests that oxidative stress contributes to the development of many human chronic diseases, including cardiovascular and neurodegenerative pathologies, metabolic syndrome, type 2 diabetes and cancer. GA and its derivative, methyl-3-*O*-methyl gallate (M3OMG), possess physiological and pharmacological activities closely related to their antioxidant properties. This paper describes the antidepressive-like effects of intraperitoneal administration of GA and two synthetic analogues, M3OMG and P3OMG (propyl-3-*O*-methylgallate), in balb/c mice with post-stroke depression, a secondary form of depression that could be due to oxidative stress occurring during cerebral ischemia and the following reperfusion. Moreover, this study determined the *in vivo* antioxidant activity of these compounds through the evaluation of superoxide dismutase (SOD) and catalase (Cat) activity, thiobarbituric acid-reactive substances (TBARS) and reduced glutathione (GSH) levels in mouse brain. GA and its synthetic analogues were found to be active (at doses of 25 and 50 mg/kg) in the modulation of depressive symptoms and the reduction of oxidative stress, restoring normal behavior and, at least in part, antioxidant endogenous defenses, with M3OMG being the most active of these compounds. SOD, TBARS, and GSH all showed strong correlation with behavioral parameters, suggesting that oxidative stress is tightly linked to the pathological processes involved in stroke and PSD. As a whole, the obtained results show that the administration of GA, M3OMG and P3OMG induce a reduction in depressive symptoms and oxidative stress.

## 1. Introduction

**Figure 1:**
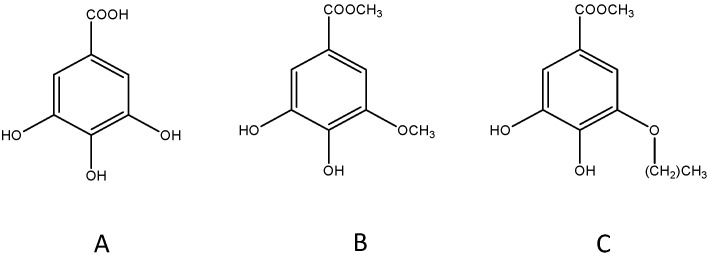
Chemical structure of gallic acid (**A**); methyl-3-*O*-methylgallate (**B**); and propyl-3-*O*-methylgallate (**C**).

Gallic acid (3,4,5-trihydroxybenzoic acid, GA, Figure 1A) is a secondary metabolite of plants, mainly formed from 3-dehydroshikimic acid through the shikimic acid pathway occurring in all plants. Thus, it is found in a wide array of foods in varying amounts according to plant species and environmental factors [1]. The main dietary sources of GA are red fruits (*i.e.*, raspberries, blueberries, and strawberries), grapes (*Vitis vinifera* L. and *Vitis aestivalis* Michx.), wine, oak bark, and gallnuts. Many plant-based foods and beverages contain GA in its esterified form and/or as GA derivatives, such as hydrolysable tannins, in addition to its free form. Green and semi-fermented or fermented teas are the most important sources of GA in the esterified forms of catechin and epicatechin, while gallotannins are not widespread in nature but have been identified in mango (*Mangifera indica* L.) and the Leguminosae and Anacardiaceae families [2].

The positive effects on human health of GA and its derivatives have been under investigation since the 1990s and many properties have been ascribed to these compounds. In addition to its well-known antioxidant activity, GA exerts anti-inflammatory, neuroprotective, cardioprotective, nephroprotective, and anticarcinogenic activities [3,4,5,6]. Moreover, it possesses antibacterial activity against different bacteria including *Escherichia coli*, *Staphylococcus aureus*, *Pseudonomas aeruginosa* and *Klebsiella pneumonia* [7]. A recent investigation has isolated a GA n-alkyl ester, methyl-3-*O*-methyl gallate (M3OMG, Figure 1B), from the leaves of *Peltiphyllum peltatum* (Torr.) Engl. (Saxifragaceae), a rhizomatous perennial herb used as a food and as a remedy in traditional medicine. A comparative *in vitro* study of the antioxidant/prooxidant activities of GA and M3OMG has revealed that M3OMG shows antioxidant activity when compared with GA, without showing prooxidant activity [8]. Further investigations on the *in vivo* antioxidant properties of M3OMG have shown that this compound is able to exert neuroprotective and cardioprotective effects against NaF-induced oxidative stress in rat brains and in erythrocyte hemolysates, respectively [9,10]. At the molecular level, GA and M3OMG exert their protective activities through different mechanisms of action. In peripheral blood mononuclear cells and EVC-304 cells, M3OMG acts through the epigenetic regulation of the expression levels of miR-17-3p, a microRNA linked to the regulation of cellular redox status [11]. Furthermore, in different prostate cancer cell subpopulations GA and M3OMG inhibit NF-kB transcriptional factor, a protein complex that controls transcription of DNA, cytokine production and cell survival [12].

Stroke is an acute cerebrovascular event caused by a reduction in the blood supplied to the brain, resulting in brain cells death. Two main types of stroke are recognized: ischemic and hemorrhagic ones. In an ischemic stroke the reduction of blood supplied to the brain is caused mainly by thrombosis due to the formation of a local blood clot causing vessels occlusion, or by embolisms due to the presence of an embolus elsewhere in the body, or by systemic hypoperfusion. The main consequences are local hypoxia, ATP decrease, and intracellular calcium increase, leading to irreversible cells damage. On the other hand, hemorrhagic stroke occurs when a weakened blood vessel ruptures. Two main types of weakened blood vessels are at the basis of hemorrhagic stroke: aneurysm and arteriovenous abnormalities, both causing bleeding into the brain after rupture. Stroke represents the second leading cause of disability in Europe and the sixth leading cause worldwide [13]. Five minutes after the onset of ischemic stroke, neurons begin to die due to oxygen and glucose deprivation, which leads to a series of negative mechanisms resulting in damage to the brain tissue. These mechanisms include oxidative stress, mitochondrial dysfunction, neuroinflammation, activation of glutamate receptors, and reduction of circulating levels of nitric oxide [14,15].

Among the complications of ischemic stroke, post-stroke depression (PSD) has high clinical relevance. One third of patients experience depression within the first year after the onset of stroke, which persists for over 20 months in 34% of elderly patients with acute stroke [16]. PSD is linked to worsened cognitive and physical outcomes, since it delays the recovery process and reduces the effect of therapy and rehabilitation. Morbidity and mortality are highly increased as a consequence. The common therapeutic approach for PSD consists of antidepressant pharmacotherapy, which is effective in increasing the number of patients reaching partial or full independence. Nevertheless, antidepressant drugs can cause side and adverse effects serious enough to make patients stop taking the medication, thus aggravating morbidity [16].

The World Health Organization reports that low fruit and vegetable intake is among the six main risk factors for cardiovascular diseases (with high blood pressure, high blood glucose, physical inactivity, overweight and obesity). Moreover, an insufficient intake of fruit and vegetables is estimated to contribute to around 9% of stroke deaths worldwide [17]. In addition, there is substantial evidence regarding the role of oxidative stress in the pathogenesis of both ischemic stroke and depression. Thus, our research group postulated the hypothesis that polyphenols and vegetable foods could have a potential therapeutic role in PSD due to their antioxidant activity [18]. Our previous research highlights the *in vivo* cardio- and neuroprotective effects of GA and its derivatives against oxidative stress [9,10,19,20]. Moreover, the benefits of GA on the brain and cognitive functions are well documented. In fact, GA reduces chronic cerebral hypoperfusion in rats and has a significant protective effect on brain cell viability [21]. In addition, GA shows antidepressive-like activity in a mouse model of unpredictable chronic mild stress [22]. A further study confirms this property by treating mice with GA brain targeted nanoparticles, which are able to improve both its antioxidant and antidepressant-like activity [23]. The antidepressive-like activity of GA has not been explored in a mouse model of PSD to date, and in view of this, the first aim of the present paper is to study the antidepressive-like effects exerted by intraperitoneal administration of GA in balb/c mice with post-stroke depression. Recent investigations into the protective effects of polyphenols have been accompanied by an increasing evaluation of their synthetic and semisynthetic analogues, which can ameliorate the bioavailability and the metabolic conversion of the original natural product, whilst maintaining both efficacy and low toxicity levels [24]. In view of this, the second aim of the present research is to evaluate the antidepressive-like activity and the *in vivo* antioxidant activity of two GA synthetic derivatives, to verify if some structural changes (*i.e.*, increase of lipophilicity) can alter the activities of these GA derivatives. The synthetic analogues, never tested on any animal model of depression, are M3OMG, previously studied by our research group regarding its *in vivo* antioxidant effect and *in vitro* epigenetic potential, and propyl-3-*O*-methylgallate, P3OMG, whose biological activities have not yet been investigated (Figure 1C).

## 2. Materials and Methods

### 2.1. Reagents and Materials

Methyl gallate, borax, dimethyl sulphate, dipropyl sulphate, sodium hydroxide, sulphuric acid, chloroform, sodium chloride and sodium sulphate were purchased from Sigma-Aldrich (St Louis, MO, USA) for the synthesis of M3OMG and P3OMG. Bovine serum albumin and a kit for protein measurement were purchased from ZiestChem Company (Tehran, Iran). 5,5-dithiobis(2-nitrobenzoic acid), ethylenediaminetetraacetic acid, nitro blue tetrazolium chloride, potassium dihydrogen phosphate, reduced glutathione, sodium dihydrogen phosphate, trichloroacetic acid, thiobarbituric acid, hydrogen peroxide, sodium carbonate, hydroxylamine chloride, ketamine, lidocaine, xylazine were purchased from Sigma-Aldrich Chemical Company, (St. Louis, MO, USA). Other chemical reagents and solvents were of analytical grade or pure and were purchased from Merck Chemical Company (Darmstadt, Germany).

### 2.2. M3OMG and P3OMG Syntheses

The synthesis of M3OMG and P3OMG was performed as reported by Nabavi *et al.* [9,10]. In brief, an aliquot of 10 g of methyl gallate was added to a mixture of borax (80 g) and water (800 mL) undergoing stirring for 30 min. Using a dropping funnel, dimethyl sulphate (30 mL) and dipropyl sulphate (30 mL) and NaOH (13 g in 50 mL water) were added dropwise from two sides of the reaction flask over 2.5 h, to synthetize M3OMG and P3OMG, respectively. After leaving the reaction mixtures to be stirred overnight, concentrated sulphuric acid (50 mL) was added. The mixtures were further stirred for 1 h and submitted to liquid extraction five times with CHCl3 (1 L). The combined chloroform extracts were washed with 500 mL of brine (26% NaCl) and dried over anhydrous sodium sulphate. Removal of the solvent under reduced pressure gave pure M3OMG and P3OMG, which were used for the *in vivo* pharmacological tests.

### 2.3. Animals

Five-week old male balb/c mice weighing 20–25 g, purchased from the Pasteur Institute of Iran, were used in the study. All mice were kept in the animal room at 24 ± 2 °C under a 12/12 h light/dark cycle and 60% ± 5% humidity. Food and water were provided *ad libitum*. All mice were allowed to acclimatize with the testing room for 24 h prior to behavioral examinations. In this study, behavioral examination was performed between 10:00 a.m. and 2:00 p.m. The animal experiments were processed following internationally accepted ethical guidelines for the care of laboratory animals in accordance with Principles of Laboratory Animals Care (NIH Publication No. 85-23, revised 1996). The ethical approval number is “81/021, 10 July 2002”.

### 2.4. Stroke Inducing

For the induction of ischemic stroke, mice were anaesthetized through the intraperitoneal administration of a mixture of ketamine (60 mg/kg) and xylazine (5 mg/kg). Bilateral common carotid artery occlusion (BCCAO) was performed as per the standard experimental animal model of ischemic stroke. In brief, both right and left carotid arteries were selected and clamped for 5 min by vascular clamps (time of ischemia). Thereafter, the vascular clamps were removed for the next 10 min (time of reperfusion), and both carotid arteries were subsequently clamped again for 5 min. Finally, the vascular clamps were removed and blood circulation was allowed to return in both carotid arteries. The surgical incisions were sutured and anaesthetized with lidocaine solution as a local anesthetic drug and the area was washed with an antiseptic solution. All mice were transported to an individual cage, kept at the standard temperature and allowed to recover normal body temperature. Rectal temperature was checked every day and animals at 37 ± 1 °C were used in the study. In addition, we eliminated animals with abnormal behavior, diarrhea or seizures [25]. 

### 2.5. GA, M3OMG and P3OMG Administration

Animals were randomly divided into 8 groups of 10 animals each. GA, M3OMG and P3OMG were intraperitoneally administered in two doses (25 and 50 mg/kg body weight) for a week. After the last application, depressive-like behaviors of all animals were tested using despair swimming and tail suspension tests. 

### 2.6. Examination of Stroke-Induced Anhedonia

For the examination of stroke-induced anhedonia, water bottles were removed from animal cages for a period of 6 h. Thereafter, two bottles were provided to each animal cage. The first bottle was filled with sucrose solution (2%, % *w*/*v*) and the second bottle was filled with water. This test is based on the evaluation of the volumes of consumed sucrose and water. Total volumes of sucrose solution and water were recorded over a period of 6 h [26].

### 2.7. Despair Swimming Test (DST)

The despair swimming test is one of the most common animal models for the examination of depressive-like behaviors. In brief, mice were individually placed in an open cylinder (25 cm height and 10 cm diameter) containing fresh water at 24 ± 2 °C (19 cm of height). The animals were forced to swim for a period of 6 min, with times recorded for periods of immobility (the period during which animals have no horizontal movement, merely keeping their head above the water surface), climbing (the period of active vertical movement, in which animals try to keep their forelegs above the water surface) and swimming (the period of horizontal movement in which animals cross the water surface) [27].

### 2.8. Tail Suspension Test (TST)

The tail suspension test is another common model for the examination of depressive-like behaviors in experimental animals. In brief, mice were suspended at a height of 58 cm for a period of 5 min through the use of adhesive tape attached 1 cm from the tail tip of each animal. The time of immobility, defined as the amount of time spent motionless during the 5 min test, was recorded [25].

### 2.9. Anesthesia and Tissue Collection

At the end of the experimental period, the mice were anesthetized by the intraperitoneal administration of ketamine (60 mg/kg) and xylazine (5 mg/kg) after withholding food for 12 h. The brain was removed and kept at −60 °C prior to biochemical assessment.

### 2.10. Preparation of Tissue Homogenate

The whole brain tissue of each animal was homogenized in 100 mM phosphate buffer saline (1:10, % *w*/*v*) containing ethylenediaminetetraacetic acid (1 mM, pH 7.4) and centrifuged (12,000 g, 30 min, 4 °C). The supernatant was separated and used for biochemical analysis.

### 2.11. Measurement of Protein Content

The protein content of the homogenates of brain was determined using the Bradford method with bovine serum albumin as the standard [28].

### 2.12. Estimation of Lipid Peroxidation

Lipid peroxidation, expressed as thiobarbituric acid-reactive substance (TBARS) formation, was determined with the method used by Di Lorenzo *et al.* [26]. Brain tissue homogenates containing 1 mg protein were mixed with trichloroacetic acid (1 mL, 20%) and thiobarbituric acid (2 mL, 0.67%) and then incubated for 1 h at 100 °C. After cooling, the precipitate was removed by centrifugation. The absorbance of the reaction mixtures was measured at λ = 532 nm using a blank containing all the reagents with the exception of tissue homogenates.

### 2.13. Determination of Superoxide Dismutase Activity

Superoxide dismutase (SOD) activity was examined according to the method used by Misra and Fridovich [29]. The reaction mixtures consisted of sodium carbonate (1 mL, 50 mM), nitroblue tetrazolium (0.4 mL, 25 μM), and freshly prepared hydroxylamine hydrochloride (0.2 mL, 0.1 mM). The reaction mixtures were mixed by inversion followed by the addition of clear supernatant of homogenates of brain tissue (0.1 mL, 1:10, % *w*/*v*). The change in absorbance of the reaction mixture was recorded at λ = 560 nm.

### 2.14. Determination of Catalase Activity

The enzyme catalase converts hydrogen peroxide into oxygen and water. Catalase activity was measured using the method described by Nabavi *et al.* [30]. The tissue homogenates (containing 5 µg of protein) were mixed with hydrogen peroxide (2.1 mL, 7.5 mM) and a time scan was performed for 10 min at λ = 240 nm and 25 °C. The disappearance of peroxide due to catalase activity was observed. One unit of catalase activity is defined as the amount of enzyme that reduces 1 µmol of hydrogen peroxide in a minute.

### 2.15. Determination of Reduced Glutathione Level

Reduced glutathione (GSH) level was determined with Ellman’s method [31]. Brain tissue homogenates (720 µL) were double diluted and trichloroacetic acid (5%) was added to precipitate their protein content. After centrifugation (12,000 × *g*, 5 min) the supernatant was taken, 5,5-dithiobis 2-nitrobenzoic acid solution (Ellman’s reagent) was added and the absorbance of the reaction mixture was measured at λ = 417 nm. A standard curve was drawn using known levels of reduced glutathione solution. Using this standard calibration curve, reduced glutathione levels were calculated for the homogenates.

### 2.16. Statistical Analysis

Statistical analysis was carried out with the SPSS statistical software package version 21.0 (SPSS Inc., Chicago, IL, USA).Results were expressed as means ± SD, and *p* < 0.01 was considered statistically significant. A Shapiro-Wilk W-test was applied to assess the normal distribution of the data. The statistical significance of the data was assessed by one-way variance analysis. When significant differences were found, Bonferroni *post hoc* testing was used to determine the differences between the groups involved. Possible bivariate correlations between different parameters were analyzed. Variables were also adjusted for multiple linear regression models in order to evaluate the association between enzyme activities (SOD and Cat), the marker of lipid peroxidation (TBARS), and the content of GSH, with each of the registered behavioral parameters.

## 3. Results and Discussion

**Figure 2:**
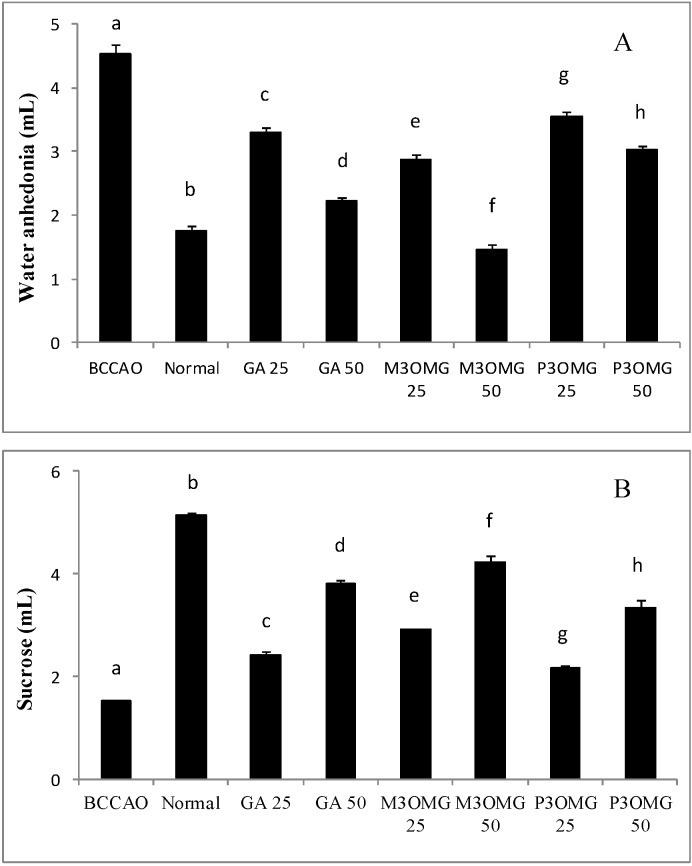
Volume of water (**A**) and sucrose solution (**B**) consumption in the stroke-induced anhedonia model. Data are shown as a mean (mL) ± SD (*n* = 3); different letters indicate statistically significant differences (*p* < 0.01) between the two groups.

In the initial phase of the investigation, M3OMG and P3OMG were synthesized as reported in the Materials and Methods section [11]. Their purity was estimated at over 95% (See Supplementary Materials). Then, GA, M3OMG, and P3OMG were studied for their *in vivo* antidepressant-like and antioxidant activities. Experimental animals were divided into three major groups: (a) a control group of healthy mice; (b) a BCCAO group of animals, which underwent bilateral common carotid artery occlusion (BCCAO); (c) 6 groups treated with GA, M3OMG and P3OMG at two different doses (25 mg/kg and 50 mg/kg). As previously demonstrated [25], bilateral common carotid artery occlusion caused anhedonia, a representative symptom of acute ischemic stroke. In fact, the results reported in Figure 2 show that the BCCAO group was characterized by a significant (*p* < 0.01) increase in water consumption and a significant (*p* < 0.01) drop in sucrose solution consumption compared to the normal group, demonstrating the validity of this animal model in the study of ischemic stroke.

Intraperitoneal administration of GA, M3OMG and P3OMG significantly (*p* < 0.01) modified water and sucrose solution consumption in a dose-dependent manner at both dosages tested, producing an improvement in the anhedonia state when compared with the BCCAO group (Figure 2A,B, respectively). The largest improvement in anhedonia was provided by M3OMG at both doses, which was able to restore normal water consumption at the 50 mg/kg dose. The least active compound was found to be P3OMG: at the highest dose (50 mg/kg) it showed the same activity (*p* = 0.033) on water consumption as M3OMG administered at the lowest dose (25 mg/kg).

**Figure 3:**
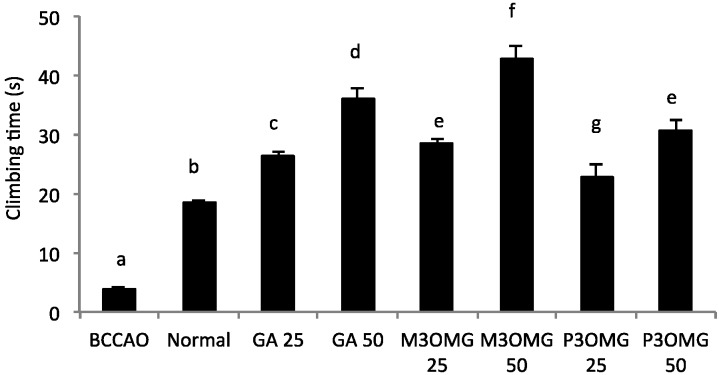
Effects of intraperitoneal administration of GA, M3OMG and P3OMG on climbing time in the despair swimming test. Data are a mean (s) ± SD (*n* = 7); different letters indicate statistically significant differences (*p* < 0.01) between the two groups.

**Figure 4:**
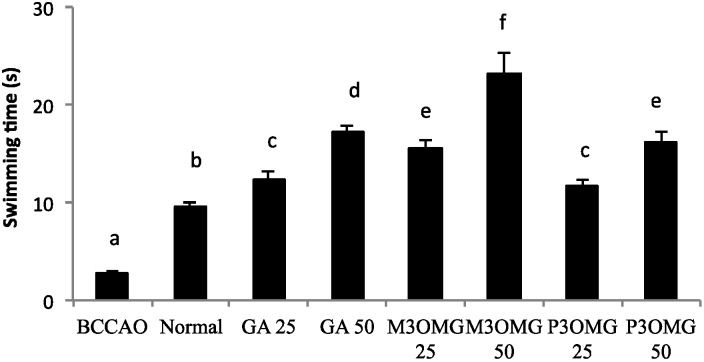
Effects of intraperitoneal administration of GA, M3OMG and P3OMG on swimming time in the despair swimming test. Data are a mean (s) ± SD (*n* = 7); different letters indicate statistically significant differences (*p* < 0.01) between the two groups.

**Figure 5:**
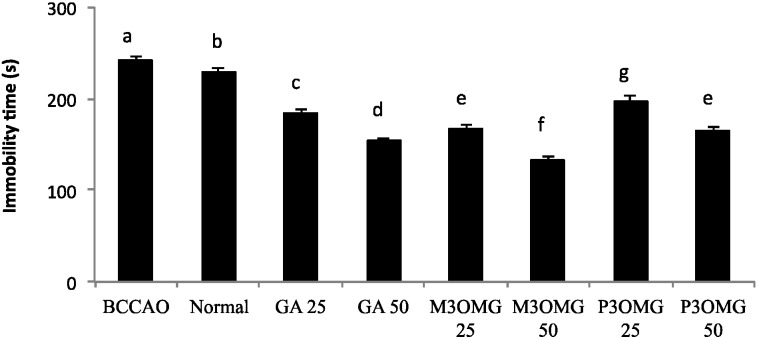
Effects of intraperitoneal administration of GA, M3OMG and P3OMG on immobility time in the despair swimming test. Data are a mean (s) ± SD (*n* = 7); different letters indicate statistically significant differences (*p* < 0.01) between the two groups.

The BCCAO animal model is also a suitable system to study depressive-like behavior through the use of two validated and commonly used tests, the despair swimming test (DST) and the tail suspension test (TST). DST is an important model of depressive-like behavior in which animals are forced to swim in a cylindrical container filled with water. Climbing, swimming and immobility periods are registered for the 6 min duration of the test [25]. At both concentrations and in a dose-dependent manner, the tested compounds showed antidepressive-like activity, significantly (*p* < 0.01) increasing the climbing and swimming times and decreasing the immobility time with respect to the BCCAO group (Figure 3, Figure 4 and Figure 5, respectively).

For all the parameters registered and at both doses, the most active compound was found to be M3OMG, whilst the least active was P3OMG, which here too shows the same activity (*p* ≥ 0.01) at the highest dosage (50 mg/kg) as M3OMG administered at the lowest dosage (25 mg/kg). 

**Figure 6:**
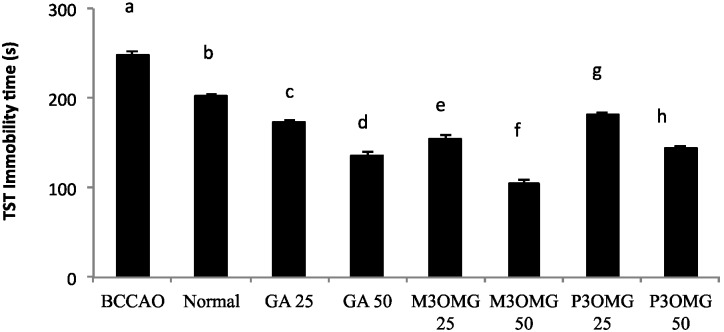
Effects of the intraperitoneal administration of GA, M3OMG and P3OMG on the tail suspension model. Data are a mean (s) ± SD (*n* = 7); different letters indicate statistically significant differences (*p* < 0.01) between the two groups.

TST describes the depressive behavior of experimental animals through the determination of immobility time in unavoidable and inescapable stress conditions. An antidepressant agent reduces the immobility times for unsuccessful attempts to escape [32]. For both concentrations and in a dose-dependent manner, GA, M3OMG and P3OMG showed high antidepressive-like activity, significantly (*p* < 0.01) decreasing the immobility time of the treated mice compared with that of the BCCAO group (Figure 6). Here too, M3OMG results as the most active compound at both doses. 

**Figure 7:**
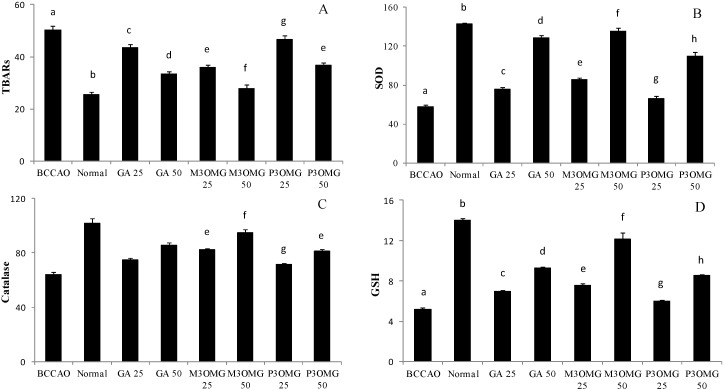
Effects of intraperitoneal administration of GA, M3OMG and P3OMG on oxidative stress levels in mouse brain tissue: TBARS levels, expressed as nmol MDA eq/g tissues (**A**); SOD activity, expressed as U/mg per protein (**B**); Cat activity, expressed as μg/mg per protein (**C**); and GSH levels, expressed as μg/mg per protein (**D**). Data are a mean ± SD (*n* = 7); different letters indicate statistically significant differences (*p* < 0.01) between the two groups.

In our previous review, we reported that there is a growing body of evidence that supports oxidative stress as playing a fundamental role in the pathogenesis of both ischemic stroke and major depression. Thus, we formulated the hypothesis that oxidative stress is also involved in the pathogenesis of post-stroke depression, and antioxidant substances could thus be useful in the treatment of this pathology [18]. Considering the *in vitro* and *in vivo* antioxidant and neuroprotective effects of GA and its related compounds, which are well documented in the literature [19,33,34,35,36], the present study was extended to the evaluation of the *in vivo* protective effect of GA, M3OMG and P3OMG against oxidative stress. The antioxidant activities of SOD and Cat, the degree of lipid peroxidation, (expressed in TBARS levels), and GSH levels were determined for mouse brains. As expected, stroke induced a significant (*p* < 0.01) increase in TBARS levels, revealing high oxidative stress in the BCCAO group. The treatment with GA, M3OMG and P3OMG significantly (*p* < 0.01) decreased TBARS levels at both tested concentrations, even though the compounds were not able to completely restore normal conditions (Figure 7A). Among the tested compounds, M3OMG was found to be the most active at both dosages (25 and 50 mg/kg), whilst P3OMG was less active. In fact, at the highest dose P3OMG showed the same activity (*p* = 0.196) as M3OMG administered at the lowest concentration. As far as endogenous antioxidant defenses are concerned, the induction of stroke was found to cause a significant (*p* < 0.01) decrease in SOD and Cat activities and GSH levels in comparison with the control group, confirming a relevant oxidative stress condition in the BCCAO group. Although the administration of GA, M3OMG and P3OMG improved endogenous antioxidant defenses, increasing SOD and Cat activities and GSH levels compared to the BCCAO group, the registered values did not attain those determined in the normal group (Figure 7B–D, respectively). M3OMG again exerted the highest antioxidant activity at both dosages, compared to GA and P3OMG, resulting as the most promising antioxidant compound with potential uses in the treatment of post-stroke depression.

To explore the relationship between the results obtained from behavioral tests and antioxidant assays in more depth, we evaluated possible bivariate correlations between the independent variables, consisting of enzyme activities (SOD and Cat), the marker of lipid peroxidation (TBARS), and the content of GSH, with the registered behavioral parameters taken as dependent variables (Table 1). Statistical significance was found for the associations of GSH, SOD and TBARS, with all the dependent variables. The highest significant associations were found between SOD and the dependent climbing and swimming variables, and for GSH and TBARS with immobility and immobility registered in TST. There were no significant associations between Cat activity and any of the studied behavioral parameters. These results support the hypothesis that oxidative stress is closely related and tightly linked to post-stroke depression.

## 4. Conclusions

In this study we have demonstrated that the intraperitoneal administration of GA, M3OMG and P3OMG restores behavioral parameters indicative of depression to healthy levels in experimental animals in which PSD has been induced by bilateral common carotid artery occlusion. M3OMG was found to be more active than GA and P3OMG, decreasing anhedonia and improving depressive-like behavior. In all behavioral tests, GA was found to be less effective than M3OMG at both doses, and P3OMG administered at the highest dose (50 mg/kg) showed the same activity as M3OMG administered at the lowest dose (25 mg/kg). These data suggest that increasing lipophilicity, moving from GA to M3OMG, increases the antidepressive-like activity while a further increase of lipophilicity, moving from M3OMG to the propyl derivative, does not correspond to an increase in activity.

The *in vivo* antioxidant activity exerted by GA, M3OMG and P3OMG supports the hypothesis that oxidative stress, which occurs during cerebral ischemia and the following reperfusion, is implicated in the pathogenesis of PSD. M3OMG shows the highest capacity to improve SOD and Cat activities, GSH levels and decrease TBARS levels, which confirms that this compound shows higher activity than GA and P3OMG. The relationship found between the results obtained from behavioral tests and antioxidant assays corroborates our hypothesis that oxidative stress is closely related and tightly linked to stroke and post-stroke depression. It is very interesting to highlight that bivariate correlations were shown for associations between GSH, SOD and TBARS, and all behavioral parameters, while no significant associations between Cat activity and any behavioral parameters were found. These last results seems to suggest that the capacity of these exogenous antioxidant compounds to modulate depressive symptoms is probably exerted primarily through SOD activity, GSH levels and a decrease in lipid peroxidation instead of through Cat activity.

Though GA and its derivatives were administered through intraperitoneal injection rather than oral ingestion, the obtained results are interesting because they show a new biological function of these hydroxy-benzoic compounds. Future studies will be performed using the oral route of administration in view of a future use for these compounds in food supplements or drugs, and to mimic the ingestion of these polyphenols as part of plant based foods and beverages. In conclusion, this work represents the first attempt to demonstrate the positive effect of GA and its synthetic derivatives on post-stroke depression and to correlate this protective activity with their antioxidant activity.

## Figures and Tables

**Table 1:** Multiple linear regression analysis on the dependent variables of climbing, swimming, immobility, and immobility in tail-suspension test (TST). *p* < 0.05 was considered statistically significant.

Antioxidant Enzymes/Marker of Lipid Peroxidation		Climbing	Swimming	Immobility	Immobility (TST)
Cat	β	0.586	0.256	−1.961	−1.730
	p	0.190	0.285	0.178	0.292
GSH	β	−7.125	−3.241	28.265	27.411
	p	0.000	0.000	0.000	0.000
SOD	β	0.340	0.135	−1.149	−1.220
	p	0.004	0.029	0.003	0.005
TBARS	β	−1.098	−0.646	4.210	5.045
	p	0.037	0.023	0.015	0.010

## Supplementary Materials

The following are available online at http://www.mdpi.com/2072-6643/8/5/248/s1.
