# Supplementary material for: Post-Stroke Depression Modulation and in Vivo Antioxidant Activity of Gallic Acid and Its Synthetic Derivatives in a Murine Model System
Source: Nutrients. 2016 Apr 28;8(5):248. doi: 10.3390/nu8050248 (PMC4882661; doi:10.3390/nu8050248)
Supplement: Supplementary file 1 [file nutrients-08-00248-s001.docx]

Supplementary Materials: Post-Stroke Depression Modulation and *in Vivo* Antioxidant Activity of Gallic Acid and Its Synthetic Derivatives in a Murine Model System

Seyed Fazel Nabavi, Solomon Habtemariam, Arianna Di Lorenzo, Antoni Sureda,
Sedigheh Khanjani, Seyed Mohammad Nabavi and Maria Daglia


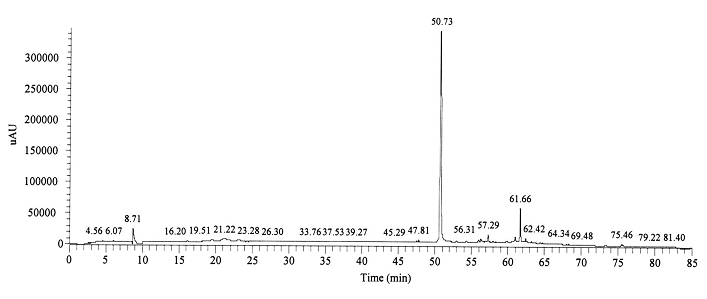


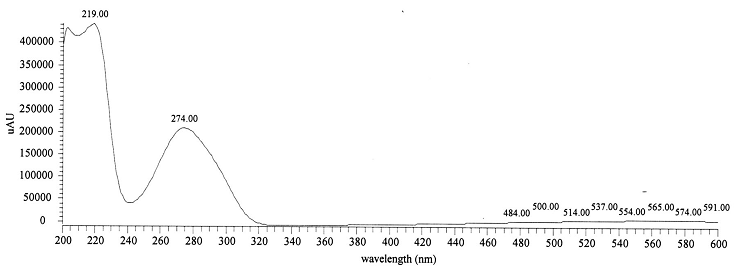


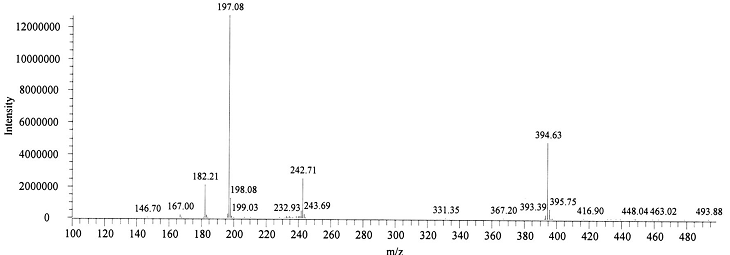


**Figure S1.** Chromatographic profile, UV-Vis spectrum and MS spectrum of synthesized M3OMG.

M3OMG analysis was performed using a Thermo Finnigan Surveyor Plus HPLC apparatus equipped with a quaternary pump, a Surveyor UV−Vis PDA detector, a LCQ Advantage Max ion trap mass spectrometer (all from Thermo Fisher Scientific, Waltham, MA, USA) through an ESI source. Separation was achieved on AERIS PEPTIDE 3.6 μm XB-C18 (250 Å 4.6 mm id, 3.6 μm) with a Ultra Cartridge C18-Peptide Security Guard column (for 4.6 mm id) both from Phenomenex, Torrance, CA, USA. The mobile phase consisted of 0.1% formic acid in water (eluent A) and methanol (eluent B) at a flow rate of 0.3 mL/min. The injection volume was 10 μL. Gradient elution was carried out using the following timetable: from 1% B to 40% B in 42 min, then to 60% B in 6 min, to 90% B in 7 min, and to 100% B in 5 min. An isocratic elution with 100% B was then carried out for ten more minutes. The resulting total run-time was 85 min, including column reconditioning. The sample tray was set at 4 °C and the column oven temperature was set at 25 °C. Spectral data were acquired in the range of 200–800 nm for all peaks. The ion trap was operated in data-dependent, full scan
(100–1000 *m*/*z*), zoom scan, and MSn mode to obtain fragment ion *m/z* with collision energy of 30% and an isolation width of 2 m/z. The negative-ion mode ESI source parameters had previously been optimized by flow injection analysis using gallic acid (10 μg/mL in 0.1% formic acid–methanol solution, 50:50, *v*/*v*) to a ionization voltage of 3.5 kV, a capillary temperature of 200 °C, a sheath gas flow rate of 45 arbitrary units, and an auxiliary gas flow rate of 20 arbitrary units. The Thermo Fisher Scientific Excalibur 2.0 software was used for data acquisition and processing.

**Figure S2.** Chromatographic profile of propylgallate and synthetized P3OMG.

The top panel represent the HPLC chromatogram of the starting material, propylgallate (Sigma Aldrich, Dorset, UK) while the lower panel shows the P3OMG HPLC chromatogram obtained from our synthesis. In both case, 20 μL of the 1 mg/mL drug solution in methanol was injected into a reverse phase column (Agilent—Eclipse Plus C18, 5 µm, 4.6 mm × 150 mm). The mobile phase was a mixture of water (A) and methanol (B). The composition of the mobile phase at a flow rate of 1 mL/min was rising from 10% to 90% B over a period of 50 min. The HPLC system was an Agilent 1200 series gradient HPLC system composed of degasser (G1322A), quaternary pump (G1322A), auto sampler (G1329A), thermostat column compartment (G1316A) maintained at 25 °C and a diode array detector (G1315D). The purity of P3OMG by HPLC was over 99%. As shown in the HPLC chromatograms, the retention time of 25.16 min obtained for propylgallate is to be shifted to 31.36 min for P3OMG suggesting the synthesis of a nonpolar and/or lipophilic antioxidant product.
